# The Glamor of Old-Style Single-Case Studies in the Neuroimaging Era: Insights From a Patient With Hemianopia

**DOI:** 10.3389/fpsyg.2019.00965

**Published:** 2019-04-30

**Authors:** Chiara Mazzi, Silvia Savazzi

**Affiliations:** ^1^Perception and Awareness (PandA) Lab, Department of Neuroscience, Biomedicine and Movement Sciences, University of Verona, Verona, Italy; ^2^National Institute of Neuroscience, Verona, Italy

**Keywords:** neuropsychology, brain lesions, perceptual awareness, consciousness, hemianopia, V1, IPS

## Single-case neuropsychology and the advent of neuroimaging

Traditionally, neuropsychology investigates the brain-behavior relationship by using a lesion-based approach. According to this approach, different brain areas subserve different cognitive processes due to the modularity of the neural system and the anatomo-functional correlation. Leaving aside the debate existing in the literature between single-case and group studies (e.g., Caramazza and McCloskey, 1988; Robertson et al., 1993), it is well-regarded that data from single-cases have proved to be very powerful in increasing our understanding on the neural correlates of cognition. In literature, there are plenty of examples of neurological patients whose unique behavior crossed the boundaries of science. This is the case of Phineas Gage whose frontal lesion led to deficits in executive functions (Damasio et al., 1994) or HM, the most thoroughly studied case of anterograde amnesia as a consequence of a temporal resection to alleviate severe epilepsy (Scoville and Milner, 1957). Other patients, though less known to the general public, had an extraordinary influence in many fields of neuropsychology. This is the case of Mr. Leborgne (known as “Tan” because that was the only word he could speak), whose behavior was fundamental for understanding language production (Broca, 1861). Further examples can be found with regards to different functions. Considering perception, for instance, we can mention studies about patients experiencing visual field defects as a consequence of occipital lesions during the First World War indicating they could complete visual forms across their blind hemifield (hemianopic completion, Poppelreuter, 1917; Riddoch, 1917), or the study of conscious and unconscious behavior while identifying objects of patient DF who developed visual agnosia following a ventral-stream damage (Goodale et al., 1991). In this context, special mention must also be made of the large body of literature on blindsight (Weiskrantz et al., 1974), with particular reference to patients FS, DB, and GY (Weiskrantz, 1986; Stoerig and Cowey, 1997; Goebel et al., 2001). The extensive study of these patients, proven to be able to unconsciously detect visual information within their blind field, contributed to uncover aspects of the visual system that cannot be highlighted under physiological conditions. All these examples have built up our knowledge not only on the correlation between the brain and its functions but also on the cognitive functions themselves, as they have been essential in the theorization of normal cognition.

The advent of neuroimaging techniques in the second half of the last century eventually resulted in a reduction of single-case studies (Chatterjee, 2005; Fellows et al., 2005; Medina and Fischer-Baum, 2017). On the one hand, the possibility to characterize brain lesions gave a strong impulse on the localization of cognitive functions in the brain, providing evidence on their anatomo-functional correlations. On the other hand, neuroimaging techniques detecting *in vivo* brain activity (e.g., PET, fMRI and more recently fNIRS) and dynamics (e.g., EEG and MEG) or interfering with normal brain processes (e.g., TMS) pushed the field toward the study of healthy participants and groups of patients. As a consequence, the number of papers on single-cases published on high-impact journals is nowadays strongly reduced (Chatterjee, 2005). At the same time, they are cited about three times less often than neuroimaging papers (Fellows et al., 2005; Medina and Fischer-Baum, 2017). Moreover, several journals previously publishing single-cases do not accept such papers any longer, except for selected cases (Medina and Fischer-Baum, 2017). Importantly, however, other high-impact scientific journals, like Cortex (Cubelli and Della Sala, 2017), have recently decided to devote a section to single case reports to counteract the reduction of single-case studies in literature given their potential value.

Here, we advocate for a renewed use of single-case studies as a valuable tool to investigate cognition by taking advantage of neuroimaging methods. The interest in single-case studies, indeed, derives from the peculiarity either of their behavior or their lesion. We trust that the combination of the lesion-based approach alongside the use of neuroimaging techniques can have a strong impact on the understanding of the brain-behavior relationship. Crucially, focal brain damages can offer the unique opportunity to test specific scientific accounts and to question theoretical models of cognition.

## The case of visual awareness: the controversial contribution of V1 and the role of the dorsal stream in accessing consciousness

One of the greatest challenges in the field of perceptual awareness is to disentangle the role of different brain areas in generating conscious experience. A still open debate exists as to whether the activity in some specific areas correlates with the content of conscious experience. Since the first observations of patients with visual field defects (Holmes, 1945), it has become evident that lesions to the primary visual cortex (V1) result in a loss of perceptual awareness in the corresponding portion of the visual field, implying a crucial role of V1 in consciousness. However, the direct contribution of V1 in conscious visual experience still remains controversial (Barbur et al., 1993; Crick and Koch, 2003; Tong, 2003; Stoerig, 2006; Ffytche and Zeki, 2011). In this respect, an influential model (Lamme et al., 1998) states that V1 becomes crucial for consciousness when receiving feedback from other areas. This advocates for a dynamical conceptualization of cortical areas involvement in consciousness suggesting that the sole feedforward activity from V1 to higher areas does not give rise to consciousness.

Another influential model, the so-called two-streams hypothesis (Goodale and Milner, 1992), postulates that visual information processed along the dorsal stream (the “vision-for-action” system), which is devoted to the transformation of visual inputs into actions, is not available to consciousness (Milner, 2012). Conversely, activity along the ventral stream (the “vision-for-perception” system), which transforms the visual input into a coherent representation of the outer world, is suitable for conscious experience. At least in the initial conceptualization of the model, V1 represents the common origin (and the only contact point) of the two streams which then diverge with the ventral stream projecting to the inferior temporal cortex and the dorsal stream projecting to the superior parietal cortex (specifically, superior parietal lobe, SPL, and intraparietal sulcus, IPS).

Within these theoretical frameworks assuming that feedback to V1 is essential for awareness and that activity in SPL/IPS remains unconscious, studying a patient with a V1 lesion would be very informative in testing the predictions of these models.

Over the last few years, we have extensively tested one hemianopic patient, SL, using several methodologies, ranging from pure behavioral measures (Celeghin et al., 2014, 2015; Mazzi et al., 2016) to EEG/ERPs (Bollini et al., 2017; Sanchez-Lopez et al., 2017; Mazzi et al., 2018b), TMS (Mazzi et al., 2014), TMS-EEG co-registration (Bagattini et al., 2015), and, very recently, fast near-infrared optical signal (Mazzi et al., 2018a).

Patient SL is a young woman suffering homonymous hemianopia on her right visual field as a result of an ischemic stroke. Structural MRI evidenced complete destruction of her left V1 (Mazzi et al., 2014). Moreover, full-field visual stimulation using fMRI did not show any activities in V1 (Celeghin et al., 2015). Accordingly, TMS at supra-threshold intensities of different portions of her lesioned occipital cortex did not result in any conscious visual percepts (Bagattini et al., 2015), thus excluding the presence of residual activity within her lesioned V1.

Having assessed that SL's V1 lesion was complete and circumscribed, we were in the privileged position to test these models. If V1 and feedback to it are necessary for awareness to emerge, as stated in Lamme's model, a complete lesion to V1 should prevent SL from having perceptual awareness in her blind field. In this respect, SL, tested with a broad variety of stimulus features (Mazzi et al., 2016; Bollini et al., 2017) reported some visual conscious experience of all kind of stimuli presented contralaterally to her lesion in a considerable number of trials (see Mazzi et al., 2017b for a review of older pieces of evidence on conscious experience within the “blind” field of hemianopic patients). Importantly, she could grade conscious visual experience within her “blind” field and the corresponding ERPs revealed differential neural activity with respect to when stimuli remained undetected (Bollini et al., 2017; Sanchez-Lopez et al., 2017; Mazzi et al., 2018b)^1^. Moreover, her electrophysiological data were similar, both in latency and topography, to those observed with healthy participants using stimuli at detection threshold level (Tagliabue et al., 2016), thus suggesting a normal pattern of neural activity even in the absence of a functioning V1.

These results show that a complete V1 lesion does not prevent from generating conscious visual experience. Instead, perceptual awareness is still possible against the predictions of Lamme's model. These data point out that V1 and feedback to it cannot be considered part of the network constituting the proper correlates of awareness (Silvanto, 2015), thus positing for a re-consideration of Lamme's model. Importantly, these data do not exclude the importance of recurrent processing among visual areas (e.g., extrastriate visual areas V2/V3 as shown by Horton and Hoyt, 1991 and Slotnick and Moo, 2003), in line with other accounts postulating the importance of synchronous activity between visual areas (Pollen, 1999; Silvanto, 2015).

With respect to the preclusion to reach consciousness for activity along the dorsal stream, it may be predicted that direct stimulation of IPS should not result in conscious visual experience. A reliable way to induce conscious visual percepts is to apply TMS over visually responsive areas in order to obtain the so-called phosphenes, i.e., conscious experience of light in the absence of light entering the eyes. To test whether the dorsal stream is part of the neural correlates of awareness, we stimulated SL ipsilesional IPS. Results showed that SL could experience reliable phosphenes rating their perceptual qualities in a manner similar to that of controls and that her conscious reports fitted well with a psychophysical detection function similar to that observed in healthy participants (Mazzi et al., 2014). Moreover, in a TMS-EEG study (Bagattini et al., 2015), we observed that SL's visual percepts induced by IPS-TMS correlated with early activity within IPS. This suggests that IPS itself can be part of the neural correlates of consciousness, at least under these conditions. Importantly, these results cannot, again, be explained by feedback activity reaching V1, as V1 is lacking in SL. Notably, analogous findings have been found with both healthy participants (Bagattini et al., 2015) and another brain-damaged patient, AM, presenting with altitudinal hemianopia on his upper visual field as a result of an ischemic stroke involving V1 (Mazzi et al., 2017a), thus suggesting a possible generalization of the results. Conversely, other authors argue for an involvement of the intact hemisphere in generating aware experience as a consequence of an ipsilesional stimulation. This is the case of patient GY since the stimulation of his ipsilesional hMT+/V5 (which is part of the dorsal stream) did not result in phosphenes perception unless accompanied by the concomitant stimulation of the homologous area in his intact hemisphere (Silvanto et al., 2007, see Bagattini et al., 2015 for a possible explanation of these contrasting results).

In sum, these results advocate for a re-consideration about the dorsal stream properties of Milner and Goodale's model. Indeed, the prediction that activity in IPS is not accompanied with perceptual experience has proved not to be satisfactory in all respects, such as when a verbal conscious report is requested, there is no time pressure in executing the command or no complex or stereotyped actions are required. However, it remains highly plausible that visuo-motor transformations need to be performed in a fast and automatic manner, thus advocating for unconscious processing. As it has recently been suggested (Milner, 2017), the two streams would not be totally segregated but the ventral and dorsal streams would communicate at higher hierarchical levels. This possibility suggests that conscious experience correlating with stimulation of IPS results from the activation of the ventral stream. This possible explanation, though, contrasts with the results on both healthy participants (Bagattini et al., 2015) and brain-damaged patients (Bagattini et al., 2015; Mazzi et al., 2017a) showing that the most likely generators of IPS-phosphenes are, indeed, IPS and not the temporal cortex. It must, however, be said that the technique used, i.e., TMS-EEG co-registration, does not possess the spatial resolution needed to conclusively localize neural activity. Future research should address this question by applying TMS over SL's IPS while concurrently recording fast near-infrared optical signal (Parks et al., 2015). This technique, indeed, is characterized by the adequate spatial and temporal resolution to record fast changes of neural activity and to assess the exact neural source of it while the patient experiences conscious percepts within her/his “blind” visual field.

Taken together, results obtained with patient SL on the involvement of both V1 and IPS in the emergence of awareness are important in the context of consciousness research. Indeed, a debate exists on the identification of which brain areas are crucial for consciousness (Dehaene and Changeux, 2011; Boly et al., 2017). The results presented here provide additional pieces of evidence in favor of the existence of a posterior cortical “hot zone,” comprising temporal, occipital and parietal cortices, as the content-specific neural correlates of awareness (Koch et al., 2016), that is the neural correlates of the subjective, phenomenal, conscious experience of the external world.

An important note of caution with respect to the role of V1 and IPS in awareness relates to the variety of results present in literature obtained with other extensively studied hemianopic patients, such as FS, GY, and DB, both across patients and across testing conditions within the same patient. With the exception of DB, which lesion extension cannot be ascertained with fMRI, FS, and GY (Stoerig et al., 1998; Goebel et al., 2001) showed no activity in V1 but only in extrastriate areas (i.e., hMT+/V5, LO, and V4/V8). These blindsight patients almost never reported conscious experience within their blind field, suggesting that activity in V1 might be essential for conscious vision whereas activity within ventral and dorsal streams is not sufficient for awareness to emerge. Importantly though, the fact that, under certain testing conditions, these patients could experience conscious percepts (Stoerig and Barth, 2001; Stoerig, 2006; Mazzi et al., 2017b) makes it essential, for future research, to deeply investigate hemianopic patients, with a variety of visual stimulation conditions and neuroimaging methods to understand how and which areas, among those thought to contribute to conscious vision, show overlapping results and which ones, instead, show only condition-specific results (Weiskrantz et al., 1995; Kleiser et al., 2001; Stoerig, 2001, Stoerig, 2006).

## Conclusions

As aptly stated by Chatterjee (2005) “a paradigmatic advance in methods is being taken for a paradigmatic shift in understanding.” The undoubtable advantages brought into the field of cognitive neuroscience by neuroimaging techniques somehow obscured the importance of extensively studying single neurological patients. However, the example of perceptual awareness given in this opinion paper with patient SL shows that results from single-case studies can bring new evidence by testing the predictions of well-regarded theoretical models. In our opinion, since this approach has proven to be helpful in one specific field, it might be helpful in general and it would make valuable advancements in scientific knowledge. We, thus, stress the need for a renaissance of the use of lesion studies, together with modern imaging techniques, as a primary tool to investigate the brain-behavior relationship.

## Author Contributions

CM and SS equally contributed to drafting and revising the final version of the manuscript.

### Conflict of Interest Statement

The authors declare that the research was conducted in the absence of any commercial or financial relationships that could be construed as a potential conflict of interest.

## Footnotes

^1^In some papers data are shown as a group but the effects are present at single subject level also.
